# The Clinical Society

**Published:** 1896-10-31

**Authors:** 


### Medical Progress and Hospital Clinics.

[*The Editor will be glad to receive offers of co-operation and contributions from members of the profession. All letters should be addressed to* The Editor, at the Office, 28 & 29, Southampton Street, Strand, London, W.C.]

### THE CLINICAL SOCIETY.

At the meeting held on October 23rd, Mr. H. H. Clutton related a case of resection of a dilated sigmoid flexure of the colon for chronic obstruction. The patient, a lady fifty years of age, had suffered from constipation as long as she remembered, and had often had attacks of pain and distension. Lately she had been much worse, and the constipation could only be relieved by copious enemata, many times repeated, and ultimately a long tube had to be used with four to five pints of water. On examination, a large, soft tumour containing flatus and fæcal fluid could always be felt in the left iliac fossa. In one of the attacks, Mr. Clutton found that eight pints of warm water could be introduced by hydrostatic pressure without much increase of pain or discomfort, and this water did not immediately come away. Meanwhile the rectum was empty. In the course of a few days the colon could, as a rule, be completely emptied, but it had latterly become increasingly difficult to obtain this result. The patient was extremely anxious for an operation, and on November 9th, 1895, Mr. Treves saw the case, and agreed as to the desirability of an attempt being made to relieve her trouble. An incision was made in the left semi-lunar line, and the distended sigmoid was easily withdrawn from the abdomen. The dilated portion terminated abruptly at each end, and the normal colon above and the rectum below could be easily approximated to each other. The sigmoid flexure was excised with about half an inch of normal bowel at each extremity, and the two ends of the bowel were united by a large Murphy's button. There was, however, some difficulty with the mesentery, and it was found that the junction was not air-tight; it was, therefore, found necessary to apply a line of Lembert's sutures round the junction. Between five and six weeks after the operation she left the home with the button still *in situ*. Beyond the anxiety caused by the presence of the button she remained in good health. The part excised resembled somewhat the normal human stomach, and it was not difficult to understand that fæcal masses would accumulate within it causing obstruction, and at the same time still further enlarging its capacity. Eleven months after the operation it was noted that she had never had any attack of obstruction, although the button was still retained and could be felt. She felt quite well, and declined any further treatment. A few days afterwards, however, she began to experience discomfort in the abdomen and some difficulty in getting the bowels to act, and this culminated in complete obstruction with immense distension. A median incision was made; the button was found lying quite free in the splenic flexure of the colon, and a stricture, which was the cause of the obstruction, was found at the seat of the first operation. The patient absolutely refused to allow colotomy to be performed, so the stricture was dealt with as in pyloroplasty, and the wound was closed. She died, however, a week after the operation.

Mr. F. C. Wallis related a case of nephrectomy for ruptured kidney. The patient, while cleaning windows, had fallen a distance of thirteen feet, alighting on an iron spike which entered the abdomen, bruising the intestines, and tearing the kidney in two pieces. The wound was enlarged, and the kidney was removed through an opening made by separating the ascending colon from its attachment. The case did well, and the patient had returned to work as a groom. Mr. Wallis thought that the majority of cases of injury to the kidney got well of themselves, but that where hæmorrhage was retroperitoneal and so severe as to cause pressure symptoms, where hæmorrhage persisted, and where there was free hæmorrhage into the peritoneal cavity, it was best to operate, and that in all cases where there was an external wound this should be explored, and dealt with accordingly.

